# Function of dynamic models in systems biology: linking structure to behaviour

**DOI:** 10.1186/2041-1480-4-24

**Published:** 2013-10-08

**Authors:** Christian Knüpfer, Clemens Beckstein

**Affiliations:** 1 Artificial Intelligence Group, University of Jena, Jena, Germany

## Abstract

**Background:**

Dynamic models in Systems Biology are used in computational simulation experiments for addressing biological questions. The complexity of the modelled biological systems and the growing number and size of the models calls for computer support for modelling and simulation in Systems Biology. This computer support has to be based on formal representations of relevant knowledge fragments.

**Results:**

In this paper we describe different functional aspects of dynamic models. This description is conceptually embedded in our "meaning facets" framework which systematises the interpretation of dynamic models in structural, functional and behavioural facets. Here we focus on how function links the structure and the behaviour of a model. Models play a specific role (teleological function) in the scientific process of finding explanations for dynamic phenomena. In order to fulfil this role a model has to be used in simulation experiments (pragmatical function). A simulation experiment always refers to a specific situation and a state of the model and the modelled system (conditional function). We claim that the function of dynamic models refers to both the simulation experiment executed by software (intrinsic function) and the biological experiment which produces the phenomena under investigation (extrinsic function). We use the presented conceptual framework for the function of dynamic models to review formal accounts for functional aspects of models in Systems Biology, such as checklists, ontologies, and formal languages. Furthermore, we identify missing formal accounts for some of the functional aspects. In order to fill one of these gaps we propose an ontology for the teleological function of models.

**Conclusion:**

We have thoroughly analysed the role and use of models in Systems Biology. The resulting conceptual framework for the function of models is an important first step towards a comprehensive formal representation of the functional knowledge involved in the modelling and simulation process. Any progress in this area will in turn improve computer-supported modelling and simulation in Systems Biology.

## Background

The modelling of complex biological systems is faced with huge challenges. New high-throughput experimentation generates enormous amounts of data which forms the empirical basis for dynamic models in Systems Biology (for short called "bio-models" in this paper). The management and analysis of the "data iceberg" [1] is impossible without computer support. Modelling and simulation on the systems level requires the integration of data from different sources on various levels in a collaborative manner [2] and the incorporation of existing models. See [3] for a detailed discussion of the challenges for modelling and simulation in Systems Biology.

Bio-models are mathematical descriptions of biological processes which are used to answer biological questions. Generally, these questions are causal questions asking for mechanistic explanations of dynamic biological phenomena. In order to serve as mechanistic explanations it is necessary that the temporal behaviour of a bio-model can be simulated by means of computers. Therefore, the bio-model has to be encoded in an appropriate computer-understandable format. System-level understanding of biological phenomena requires the integration of bio-models from different abstraction levels expressed in different modelling paradigms [4]. Computer support for modelling and simulation is an important contribution to meet the challenges in Systems Biology. This computer support has to be based on formal representations of relevant knowledge fragments. A first step towards such a formal representation is an ontological analysis of the domain of knowledge in question. The result of such an analysis will be a "conceptual framework", i.e. a set of categories and relations between them which can be used to capture the respective knowledge. What we call a "conceptual framework" is usually called "domain model" or "conceptual model" in computer science. In the context of this work the use of the traditional terms seems to be misleading because the omnipresent term "model" is used with different meaning.

The function of a bio-model describes its role in the scientific process of finding mechanistic explanations for biological phenomena. Beside this teleological aspect of a model’s function there are pragmatical aspects: The function of a bio-model also describes the use of the model in simulation experiments. In such simulation experiments behaviours of the model are generated. Thus, function links a model (structure) to its behaviours. To summarise, the function of bio-models describes why and how to use models in simulation experiments: 

"Models can fulfil many functions as we have seen; but they generally perform these functions not by being built, but by being used. Models are not passive instruments, they must be put to work, used, or manipulated." ( [5], p.32)

Physiology is the branch of biology dedicated to function of living systems. The notion of "function" as used in physiology incorporates two aspects of a biological entity: (1) The function states a role an entity plays as a component of an encompassing process. The biological function is therefore tied to a specific process. (2) The function characterises the behaviour which the entity has to exhibit for fulfilling its role. Biological function links system structure (the entities and relation) to behaviour. The most famous example is the function of genes which links the genotype to the phenotype. In this paper we transfer this notion of function to bio-models.

**Figure 1:**
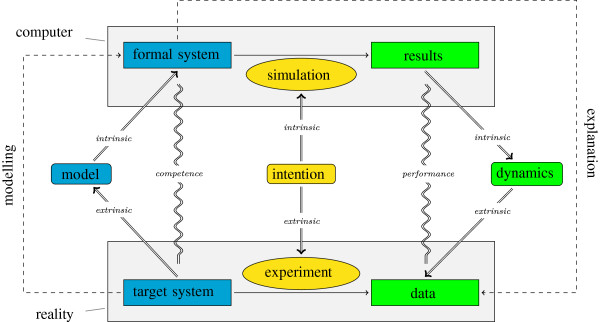
**Structure, function and behaviour of a bio-model.** Structure (blue/left), function (yellow/middle) and behaviour (green/right) of a bio-model. The model relates the (intrinsic) computer representation with the (extrinsic) biological reality. (1) Structure: The biological target system is transferred into a model which can in turn be intrinsically interpreted as a formal system. This establishes a modelling relation between the two systems. If there is a valid mapping between the components of the target system and the formal system, we call the model a competence model. (2) Function: The intention of the model is its use in simulation experiments for explaining biological phenomena observed in biological experiments. (3) Behaviour: The simulation experiments produce results which can be interpreted as the dynamics of the model. This dynamics can be related to the interpreted data of the biological experiments. If the behaviour of the model is similar to the behaviour of the biological system, we call the model a performance model with respect to the corresponding biological phenomena. Explanation is using a competence model in an simulation experiment which makes it a performance model with respect to the biological phenomena to explain.

We claim that the function of a bio-model links its structure to its behaviour. Before we describe the function of a bio-model in detail we will introduce the "meaning facets" which provide a framework to systematically describe the knowledge involved in creating and using bio-models. In the meaning facets framework the function of a bio-model mediates between its structure and its behaviour (cf. Figure 1). The present paper focuses on how the function plays this mediating role.

In order to do so, we first analyse the function of bio-models and develop a conceptual framework. Then, the conceptual framework is used to show whether and how the function can be formalised. Thereby, we review existing formal approaches and identify missing pieces. For one of the identified gaps we outline a possible filler. Finally, we compare our conceptual framework with related work.

### Meaning facets of bio-models

Generally, a mathematical model establishes a relation between a system under observation (what Rosen [6] calls the "natural system") and a formal system. Rosen calls this the "Modeling Relation". In order to be useful the modelling relation has to be an isomorphism: the structure of the formal system, expressed as entities and relations between them, can be mapped onto the structure of the natural system, recognised as objects and interactions.

In our analysis of the modelling process in Systems Biology we make two important observations about bio-models [7]. First, a bio-model has a dual interpretation: In order to be used in computer simulations the encoded model has to be *intrinsically* interpreted with respect to the encoding format used. This can be done without referring to the modelled natural system. Furthermore, a model has to be related to the natural system (cf. Rosen’s modelling relation [6] mentioned above), i.e. it has to be *extrinsically* interpreted. Second, dynamic models are considered on three levels: Models are systems of components and relations (*model structure*). Models are used in simulation experiments for answering biological questions (*model function*). Models exhibit temporal changes (*model behaviour*). The three levels of bio-models are inspired by function modelling in engineering (see, e.g., [8]). In function modelling formally represented teleological knowledge is used for the design and diagnosis of engineering artefacts.

We claim that full computer support of the overall modelling process has to be based on a complete description of a bio-model encompassing all six "meaning facets" [9], i.e. the intrinsic and extrinsic sides on each of the three levels. If we look at the scientific process of modelling a biological system in order to explain data observed in experiments we can clearly identify the three levels and the dual interpretation (see Figure 1). More details about the six meaning facets are given by Knüpfer et al. [9] including a complete description of an example model with respect to the meaning facets. However, in this paper we will focus on the functional facets of bio-models.

## Conceptual framework for the function of bio-models

A bio-model used in simulation experiments in order to answer biological question has an intrinsic and an extrinsic function (see above) which can be understood in teleological and in pragmatical terms. There is a third perspective on the function of bio-models, which we call "conditional": the role played by an entity depends on the context; in different situations or under different conditions an entity may have different roles. In the following we will investigate the individual functional aspects in detail. The introduced concepts were distinguished by writing them in *italics* throughout the paper. Table 1 summarises the used concepts.

**Table 1:** Functional aspects of bio-models

	**Intrinsic**	**Extrinsic**
**Teleological function**	*Intended use*	*Model use intention*
	*Constraints*	*Assumptions*
**Conditional function**	*Model instantiation*	*Boundary conditions*
	*Initial values*	*Initial state*
**Pragmatical function**	*Setup*	*Experimental settings*
	*Post-processing*	*Result calculations*

The presented conceptual framework extends the Minimum Information About a Simulation Experiment (MIASE, [10]) which is a reporting guideline for simulation experiments performed on bio-models. The rules of MIASE regard the conditional and pragmatical function of bio-models. The conceptual framework presented here also incorporates the teleological function and relates the description of simulation experiments (intrinsic function) to the biological counterparts (extrinsic function).

It should be noted that a bio-model can have more than one function if it be used to answer different biological questions. The separation of structure and function of bio-models in our meaning facets framework facilitates this re-use of the same model for different purposes. However, the model structure can restrict the adequate use of a model in so far as not every model structure is able to answer a given question.

### Teleological function

Bio-models play an important teleological role in answering questions about the biological system under investigation. These questions lead to specific intentions of using the model in simulation experiments (called "*model use intentions*" in the following). *Model use intentions* may be, for example, the explanation of observed behaviours or the prediction of possible behaviours. What is an accepted explanation or prediction depends on the scientific field and community [11]. Furthermore, there are specific *assumptions* underlying the model use intentions, e.g. the assumption that a specific reaction is very fast. The extrinsic teleological function of bio-models refers to the *model use intentions* and the underlying *assumptions*.

The *intended use* of the model in simulation experiments has to reflect the *model use intentions*. Depending on these intentions different types of simulation experiments may be appropriate. Often, different simulation experiments have to be combined in order to yield the desired outcome. *Constraints* which are in line with the *assumptions* are imposed on the simulation experiments. Such *constraints* may include value restrictions, ratios between values, and conservation rules. The intrinsic teleological function of a bio-model describes its *intended use* and imposed *constraints*.

### Conditional function

The questions addressed by the model, in general, assume certain *boundary conditions* and a specific *initial state* of the experimentally observed biological system. The *boundary conditions* determine the environment of the biological system (e.g. temperature, pH, nutrition) and may be reflected by corresponding kinetic data. It is also possible to give plausible ranges for some of the conditional values instead of single values. The extrinsic conditional function of a bio-model is expressed in terms of *boundary conditions* and *initial states*.

A bio-model contains state variables and formal parameters. In order to be used in simulation experiments, concrete values must be assigned to all parameters. This is called "*model instantiation*". Furthermore, the *initial values* for all state variables have to be chosen. The intrinsic conditional function of a bio-model makes the model ready to be used in simulation experiments by means of *model instantiation* and choice of *initial values*.

### Pragmatical function

As mentioned above, bio-models explain or predict the behaviour of the modelled biological system. This teleological function requires a complementary description of the *experimental settings* which lead to the observed behaviour and allow for the verification of the predicted behaviour. Usually the experimental data is transformed into the final observations by means of *result calculations*. The extrinsic pragmatical function of a bio-model describes the *experimental settings* and *result calculations* related to the dynamic phenomena under investigation.

Bio-models are used in simulation experiments. The *setup* of the simulation experiments precisely describes the procedure applied to the instantiated model. This involves the simulation algorithm used and specific settings for this algorithm. In addition, the exact steps, their order and applied perturbations have to be specified. *Post-processing* of the raw data from the simulation experiments generates the desired outcome. The intrinsic pragmatical function of a bio-model describes the *setup* of the simulation experiments applied to the model structure and the *post-processing* which finally produces the model behaviour.

## Formal approaches to the function of bio-models

In this section we briefly review existing approaches for formalising the different functional aspects of bio-models. The aim of this review is to investigate the coverage of formal representations of functional knowledge as a basis for computer support. Table 2 provides an overview of the formal approaches. The classification of the formal approaches in checklists, languages and ontologies is motivated by [12]. Table 2 can be seen as a detailed view of the middle column "Simulation description" of Figure 1 from ( [12], p.7) which reviews existing approaches for bio-models. The formal approaches displayed in Table 2 and the gaps are discussed in the following sections.

**Table 2:** Formal approaches for functional aspects of bio-models

**Functional aspect**		**Checklists**	**Languages**	**Ontologies**
**Teleological function**				
Intrinsic:	*Intended use*	MIASE	SED-ML	MINTENTO
Extrinsic:	*Model use intention*	*Unknown*	*Unknown*	MINTENTO
**Conditional function**				
Intrinsic:	*Model instantiation*	MIASE	SED-ML	*Not applicable*
Extrinsic:	*Boundary conditions*	MIBBI	SABIO-RK^*^	XCO
**Pragmatical function**				
Intrinsic:	*Setup*	MIASE	SED-ML	KiSAO
Extrinsic:	*Experimental settings*	MIBBI	FuGE	MMO

^*^The database scheme of SABIO-RK could be seen as a formal language for experimental conditions.

### Checklists

To start the formalisation of a specific kind of scientific data, like bio-models, experiment protocols, or experimental results, the responsible community first of all has to agree on the information needed. So-called "Minimum Information Checklists" state what information at least has to be described. Checklists are semi-formal in that they structure the information. The information, however, is still formulated in natural language.

The concrete intrinsic functional description of the simulation experiments is the main focus of MIASE [10]. MIASE requests information about the simulation type (*intended use*, teleological function), the *model instantiation* (conditional function), the exact experimental *setup*, and the necessary *post-processing* (pragmatical function).

For the description of the extrinsic function there are a lot of specific checklists listed in the MIBBI portal (Minimum Information for Biological and Biomedical Investigations, [13]), for example, MIAME (Minimum Information About a Microarray Experiment, [14]) and MIFlowCyt (Minimum Information about a Flow Cytometry Experiment, [15]). The listed checklists concern information about the *boundary conditions* (conditional function) and the *experimental settings* (pragmatical function) for specific types of biological experiments. We are not aware of any checklists for the *model use intentions* (teleological function) of bio-models.

### Languages

In most cases a checklist can be translated to a specification of a formal language. Such a formal language for describing simulation experiments is SED-ML (Simulation Experiment Description Markup Language, [16]) which allows to specify the type of simulation, i.e. the *intended use* (intrinsic teleological function), the *model instantiation* and *initial values* (intrinsic conditional function) as well as the *setup* and *post-processing* of the simulation experiments (intrinsic pragmatical function). However, SED-ML can not fully describe the *intended use* and the imposed *constraints*. The Systems Biology Markup Language (SBML, [17]) used for the encoding of the model structure is also able to determine parameter values (*model instantiation*) and *initial values*. But, in order to be able to reuse models in different simulation experiments we suggest to clearly separate descriptions of models (in SBML) from descriptions of their use (in SED-ML).

There are languages for describing extrinsic experimental conditions and specific biological experiments. SABIO-RK (System for the Analysis of Biochemical Pathways – Reaction Kinetics, [18]), for example, allows the representation of experimental and environmental *boundary conditions* for measurements of the stored kinetic data. The Functional Genomics Experiment Object Model (FuGE, [19]) describes *experimental settings* in functional genomics. We will not go into detail here. We are not aware of any languages for the *model use intentions* (teleological function) of bio-models.

### Ontologies

Ontologies formalise conceptual knowledge. They can provide vocabularies for formal languages.

The Kinetic Simulation Algorithm Ontology (KiSAO, [20]) is employed within SED-ML to precisely specify the algorithms used for the simulation experiment. Thus, KiSAO contributes to the description of the intrinsic pragmatical function of bio-models (*setup*).

Ontologies for intrinsic *model instantiation* seem not to be very useful: There is not much conceptual knowledge involved in assigning values to parameters and variables. This is not the case for the (extrinsic) *boundary conditions*. The Experimental Conditions Ontology (XCO) [21] provides a rich vocabulary of experimental conditions for phenotype experiments. The Measurement Method Ontology (MMO) [21] can be used for specifying the measurement method in descriptions of extrinsic *experimental settings*. Because XCO and MMO are slightly out of our scope we will not provide further details.

At the moment there exists no ontology for the teleological function of bio-models. In the next section we propose an ontology for both the intrinsic and the extrinsic teleological function.

## A new ontology for intentions of bio-models

In this section we provide first ideas for an ontology for the teleological function of bio-models. For convenience, we will call this proposed ontology MINTENTO (Modelling Intention Ontology). Such an ontology would, for example, facilitate computer support for the choice of appropriate simulation types and methods based on intentions. Such an ontology would also allow to place simulation results in the context of the respective intentions. As a consequence, this will ease the reuse of simulation results.

How could an ontology for the teleological function of bio-models look like? Here we present first ideas for MINTENTO. The teleological function of bio-models comprises of two aspects: the extrinsic *model use intentions* and the intrinsic *intended use* of the model fulfilling the intentions. This reflects the difference between a function (here: "model use intention") and its realisation (here: "intended use") which is also employed for the ontological analysis of biological function by Burek et al. [22]. There may exist realisedBy relations between classes of the two main MINTENTO branches. A statement of the form "*A*realisedBy*B*", where *A* is a subclass of "model use intention" and *B* is a subclass of "intended use", means for every instance *a* of A there is an instance *b* of *B* (a process) such that the execution of *b*realises*a*. As a consequence the instance level relation realises reads as "fulfils or contributes to".

In MINTENTO we want to cover both aspects and appropriate realisedBy relations between them. To grammatically reflect this distinction we use infinitive verb forms (e.g. "to construct") for *model use intentions* and nouns for the realising processes (e.g. "construction"). MINTENTO specialises the upper ontology BFO (Basic Formal Ontology, [23]): "model use intention" is a subclass of the BFO concept "snap:Function" and "intended use" is a subclass of the BFO concept "span:Process".

The *model use intentions* for the investigation of a biological system are formulated in natural language. There is a wide diversity of *model use intentions*. Morrison and Morgan describe different high-level (teleological) functions of models [5]. Summarising they state: 

"[...] models fulfil a wide range of functions in building, exploring and applying theories; in various measurement activities; and in the design and production of technologies for intervention in the world." ( [5], p.24)

**Figure 2:**
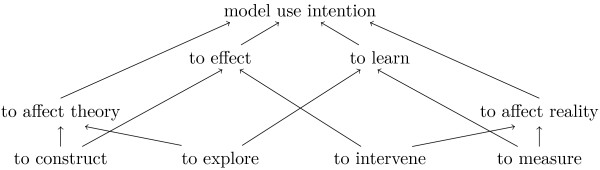
**Upper level of MINTENTO: model use intention.** Upper level terms for the "model use intention" branch of MINTENTO. The edges represent subclass (is-a) relations.

The set of ways how models can be used, provided by Morrison and Morgan [5], is a good starting point for an upper classification of general *model use intentions*. Models are used "to construct (a theory)", "to explore (a theory)", "to measure", and "to intervene". These four upper level *model use intentions* are based on two distinctions: (1) A model "can mediate between theory and the world" ( [5], p.11), i.e. the model is either intended "to affect theory" or "to affect reality". (2) There is a distinction between using a model "to effect (something)" and "to learn (something)" ( [5], p.11). Figure 2 shows the two distinctions and the resulting upper level *model use intentions*.

**Figure 3:**
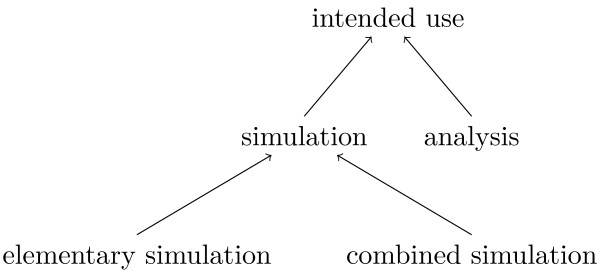
**Upper level of MINTENTO: intended use.** Upper level terms for the "intended use" branch of MINTENTO. The edges represent subclass (is-a) relations.

In this paper we focus on dynamic and computational bio-models. The *intended use* of this kind of model is the simulation of the model’s dynamic behaviour. There is also the possibility to mathematically analyse dynamic properties of a bio-model without explicitly considering any temporal behaviour. Therefore, the upper level of the "intended use" branch of MINTENTO consists of "simulation" and "analysis". Simulations are further differentiated into "elementary simulation" and "combined simulation" (see Figure 3). An elementary simulation calculates the temporal behaviour of the model for a single model instantiation whereas a combined simulation consists of several elementary simulations for different *model instantiations* and integrates the single results into the final outcome. An example for an elementary simulation is a uniform time-course simulation, where the state of the formal system specified by the model is calculated at discrete equidistant time points. This simulation type is covered by SED-ML Level 1 Version 1 [24]. A parameter scan is an example for a combined simulation where the scanned parameter is varied in the particular elementary simulations. The next SED-ML version is supposed to provide mechanisms for defining combined simulations, like sequential and nested simulations (cf. the website [25] for current developments).

In order to illustrate such realisedBy relations between *model use intentions* and *intended use* we look at some common tasks ("model use intentions"), a bio-model is used for: (1) "to approximate" observed behaviour, (2) "to investigate" the variability in behaviour, (3) "to demonstrate" the ability for specific kinds of behaviour, and (4) "to explore" parameter influence on the behaviour. Each task requires a different corresponding (realisedBy) simulation type: (1) time series (eventually including parameter fitting), (2) bifurcation analysis, (3) stability analysis, and (4) parameter scan.

## Related conceptual frameworks

In this section we will compare our conceptual framework for the function of bio-models with related conceptualisations from the fields of Artificial Intelligence, modelling and simulation, functional modelling, and biology.

The dual intrinsic/extrinsic interpretation of bio-models is rooted in the "knowledge representation hypothesis" from Artificial Intelligence: 

"Any mechanically embodied intelligent process will be comprised of structural ingredients that a) we as external observers naturally take to represent a propositional account of the knowledge that the overall process exhibits, and b) independent of such external semantical attribution, play a formal but causal and essential role in engendering the behavior that manifests that knowledge." ( [26], p.15)

Simon generalises this duality to all kinds of artifacts which serve as interfaces between an inner and an outer environment [27]. Rosen’s modelling relation [6] is a congruence between an extrinsic/outer natural system and an intrinsic/inner formal system established by a model.

As stated above, a conceptual framework provides categories and relations between them which are employed to capture relevant knowledge fragments. There are other conceptual frameworks for modelling and simulation in general [28,29], and in particular for bio-modelling [12]. Our meaning facets, however, provide much more details and are more rigid, i.e. they provide categories of finer granularity and define them more precisely. Our meaning facets are therefore a solid foundation for computer support for modelling and simulation based on formal knowledge representation.

Zeigler’s notion of an "experimental frame" [29] resembles the *model instantiation* (conditional function) and the experimental *setup* (pragmatical function) presented in this paper. His experimental frame "is the operational formulation of the objectives that motivate a modeling and simulation project" ( [29], p.27), i.e. it also describes the teleological function of a model.

The field of functional modelling relates structure, behaviour and function of engineering artifacts. Erden et al. reviews the different approaches to formalise function and its relations to structure and behaviour [8]. Two different notions of function are employed [8]: On the one hand, function is mediating between structure and behaviour and determines the "structural behaviours", i.e. all possible behaviours the model is able to show. This is essentially what we call conditional and pragmatical function, respectively. On the other hand, function refers to the intentions of the modeller and restricts the possible behaviours to the "expected behaviours". This notion of function as purpose corresponds to our teleological function. In short, functional modelling addresses "the questions of what the device and its components do or what the purpose of the device and its components are" ( [8], p.149). Joining these two sides, function becomes "the bridge between human intention and physical behavior of artifacts" ( [30], p.271). The distinction between structural and expected behaviours originates from Gero [31].

We transfer the notion of function in biology to modelling in systems biology. There are some strong parallels between function in biology and function of bio-models. The idea that function links between structure and behaviour is deeply rooted in molecular biology, as, e.g., stated by Lander: 

"If one such behavior seems useful (to the organism), it becomes a candidate for explaining why the network itself was selected, i.e., it is seen as a potential purpose for the network." ( [32], p.0712)

However, we will not discuss the notion of function in biology further here. Krohs and Kroes compare the notion of function in biology and technology and examines disanalogies and parallels [33].

There are some formal approaches to biological function. Ontologies like EcoCyc [34] and the Gene Ontology [35] list molecular functions played by biological entities. Burek et al. presents an ontology of biological functions which formalises three functional aspects [22]: the so-called "function structure", the realisation and the has-function relation, which could be related to our teleological, pragmatical and conditional function, respectively. Because of the different knowledge domains of [22] and our work, the two are not identical. We provide more details on each functional aspect. However, the similarity of these conceptualisation and the one presented in this paper can be seen as further justification for transferring the notion of biological function to bio-models.

## Conclusion

We have applied the notion of function to dynamic models in Systems Biology. Function is the link between the model structure and the model behaviour. The intrinsic function of bio-models describes three aspects of the model’s use: Why should the model be used in simulation experiments (teleological function)? Which model instance should be used in simulation experiments (conditional function)? How should the model be used in simulation experiments (pragmatical function)? The extrinsic function of a bio-model refers to the intentions of using the model in order to address biological questions.

The presented conceptual framework of the functional aspects of bio-models was used to systematise and review corresponding formal accounts. Some functional aspects are well covered by checklists, languages and associated ontologies. However, there are no checklists and languages for the extrinsic teleological function. Closing these gaps would improve the standardised descriptions of biological experiments.

At the moment there also exists no ontology for the *model use intentions* and the *intended use* of bio-models. We outlined such an ontology, the Modelling Intention Ontology (MINTENTO). Since this is ongoing work we are only presenting first thoughts about MINTENTO here. We nevertheless believe that these thoughts provide an important starting point for developing an ontology for the teleological function of bio-models. Next steps would involve the incorporation of lower levels terms and the specification of MINTENTO in some standard format like OWL (Web Ontology Language, [36]).

Our ontological analysis of functional aspects of dynamic models in Systems Biology and their use in simulation experiments provides an important prerequisite for formalising the involved knowledge. Ultimately, this will improve any computer-supported research method for answering biological questions by means of bio-models.

## Competing interests

The authors declare that they have no competing interests.

## Authors’ contributions

CK developed the meaning facets framework and analysed the different functional aspects of bio-models. CB supervised this work. Both authors read and approved the final manuscript.
